# Safety assessment of rat embryonic fraction for *in vivo* regenerative therapy

**DOI:** 10.1242/bio.060266

**Published:** 2024-08-22

**Authors:** Sivarama Prasad Darsi, Somorita Baishya, Veerababu Nagati, Kala Kumar Bharani, Satyanarayana Swamy Cheekatla, Sujesh Kumar Darsi, Adi Reddy Kamireddy, Ram Reddy Barra, Ashok Kumar Devarasetti, Sreedhar Surampudi, Jayaram Reddy Singireddy, Siva Kumar Kandula, Anil Kumar Pasupulati

**Affiliations:** ^1^ Department of Biotechnology, School of Life Sciences, Gitam University, Visakhapatnam, AP, India 530045; ^2^ Department of Biochemistry, University of Hyderabad, Hyderabad, TG, India 500046; ^3^ Department of Veterinary Pharmacology and Toxicology, P.V. Narasimha Rao University of Veterinary Sciences, Rajendra Nagar, TG, India 500030; ^4^ Department of General Medicine, ESI Corporation, Gunadala, Vijayawada, AP, India 520004; ^5^ Department of Internal Medicine, Banner Health Center, Maricopa, AZ, USA 85138; ^6^ Department of Physiology, Apollo Institute of Medical Sciences and Research, Hyderabad, TG 500090, India; ^7^ Department of Veterinary Biochemistry, P.V. Narasimha Rao University of Veterinary Sciences, Mamnoor, Warangal, TG, India 506166; ^8^ Department of Biochemistry, Aware College of Medical Lab Technology, Bairamalguda, Hyderabad 500035, India; ^9^ Department of Urology, Hyderabad Kidney & Laparoscopic Centre, Malakpet, Hyderabad, TG 500036, India

**Keywords:** Embryonic protein fraction, Regenerative therapy, Immunogenicity, Tumorigenicity, Proteomic analysis

## Abstract

Regenerative therapy is considered a novel option for treating various diseases, whereas a developing embryo is a prime source of molecules that help repair diseased tissue and organs. Organoid culture studies also confirmed the inherent biological functions of several embryonic factors. However, the *in vivo* safety and efficacy of embryonic protein fraction (EPF) were not validated. In this study, we investigated the effectiveness of EPF on healthy adult rats. We obtained embryos from Sprague-Dawley (SD) female rats of E14, E16, and E19 embryonic days and collected protein lysate. This lysate was administered intravenously into adult SD rats on sequential days. We collected blood and performed hematological and biochemical parameters of rats that received EPF. C-reactive protein levels, interleukin-6, blood glucose levels, serum creatinine, blood urea, total leucocyte counts, and % of neutrophils and lymphocytes were comparable between rats receiving EPF and saline. Histological examination of rats' tissues administered with EPF is devoid of abnormalities. Our study revealed that intravenous administration of EPF to healthy adult rats showed that EPF is non-immunogenic, non-inflammatory, non-tumorigenic, and safe for *in vivo* applications. Our analysis suggests that EPF or its components could be recommended for validating its therapeutic abilities in organ regenerative therapy.

## Teaser

**Summary:** Rat embryonic protein fraction from E14th, E16th, and E19th day did not elicit adverse effects in healthy adult rats. These data suggest components of EPF could be explored for regenerative therapy.

## INTRODUCTION

The current therapy of transplantation of intact tissues or organs suffers from several obstacles, including the scarcity of donor supply and severe immune complications. The loss of tissues and organs during injury or disease inspires the development of therapies that can regenerate tissues and decrease reliance on organ transplantations (Mao and Mooney, 2015). Regenerative medicine can repair tissues damaged by age, disease, or trauma and normalize congenital deformities. The canonical regenerative strategies suffer from several obstacles (Mao and Mooney, 2015; Oh et al., 2014). Stem cells isolated from adult tissue or induced require tight control over their behavior following transplantation. Imparting complete vasculature for replacement tissues to be anastomosed with host vessels is crucial for graft survival. Ensuring complete vasculature for grafting tissue is challenging. In addition, a greater understanding of the donor and recipient immune system's role in regeneration is needed to achieve a desirable immune response. Therefore, there is an increased demand for alternative approaches to regenerative medicine therapies.

Regenerative medical research has stumbled into a new direction in using embryonic protein fraction (EPF). The tissue differentiation, organogenesis, and growth dynamics are strictly operated through specific embryonic factors (Keller, 2005). During the development of an embryo, tissue differentiation, and organogenesis are manifested with embryonic differential expressions of the transcriptome (Golubeva and Symer, 2014) and proteome (Nagano et al., 2005). Dihazi et al. performed a comparative proteomic analysis of rat embryonic kidneys from E14, E16, E19, and P1 stages. When EPF was subjected to 2D electrophoresis, 977 spots were identified, and 288 were non-redundant proteins. Functional annotation of the identified proteins revealed that they are involved in kidney development in stage-specific pathway activation, and several proteins in EPF have regenerative functions (Dihazi et al., 2015). Studies suggest that embryonic cell transcriptome differs from adult cell transcriptome (Ulloa-Montoya et al., 2007). Thus, the embryo is a reservoir of different proteins that might be involved and can be used to regenerate or repair damaged organs in adults.

One of the prominent studies is transforming adult somatic cells into induced pluripotent stem cells using the Yamanaka factors Oct3/4, Sox2, Klf4, and c-Myc. All these factors are exclusively expressed on the 14th day of the mice embryo (Liu et al., 2008). Another study has confirmed that early nephrogenesis is due to epigenetic factors like Cbx1, Cbx3, Cbx5, and Trim-28 isolated from developing kidneys (Dihazi et al., 2015). While supplemented with these factors, cells will be differentiated into ureteric buds and elongated into renal vesicles, forming comma- or S-shaped bodies. In another study, proteins from vesicles of amniotic fluid stem cells are injected through the intra-ventricular route; these injected cells are homed in the damaged kidney and exert a renoprotective function (Lewis et al., 2018). All these studies have encouraged the therapeutic application of embryonic molecules treating injured or diseased organs in adults. Therapeutically, embryonic molecules may have an advantage over stem cell therapy, as stem therapy has crucial limitations such as tumorigenicity (Ben-David and Benvenisty, 2011), heterogeneity (Yamanaka, 2020), and immunogenicity (Zhao et al., 2011). In the present study, we isolated EPF from pregnant rats and evaluated its efficacy in adult rats. We assessed the safety of EPF in adult rats by administering EPF intravenously and monitored tumorigenicity and immunogenicity in adult rats.

## RESULTS

### Embryonic fraction did not alter the biochemical parameters in healthy adult rats

**Fig. 1.**
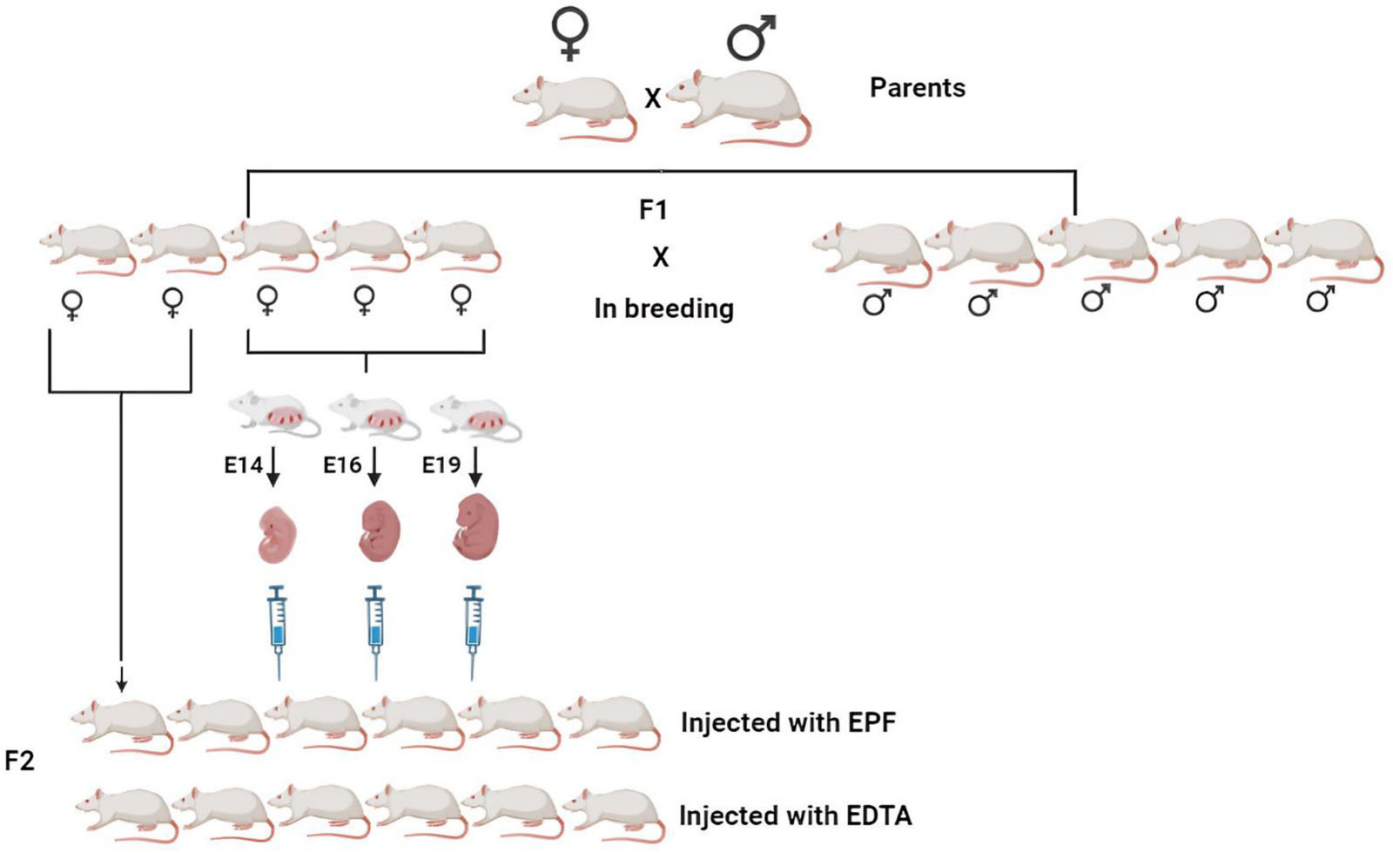
**Experimental procedure.** Eight-month-old male and female SD rats were allowed to mate to obtain F1 generation pups. In the F1 population, five males and five females were allowed to mate, and mating was confirmed by vaginal swab microscopy and vaginal plug formation. Three pregnant rats were euthanized on three different embryonic days (E14, E16, and E19) to isolate embryos, and were processed to prepare embryonic protein fractions (EPF). The other two pregnant rats were allowed to deliver pups and they were raised for up to 13 months. F2 rats were separated into two groups: control and treatment (*n*=6 each). Six of these F2 rats were injected with EDTA, whereas the remaining six were injected intravenously with E14, E16, and E19 day EPF serially with a gap of 3 days (scheme was created using Biorender).

**Fig. 2.**
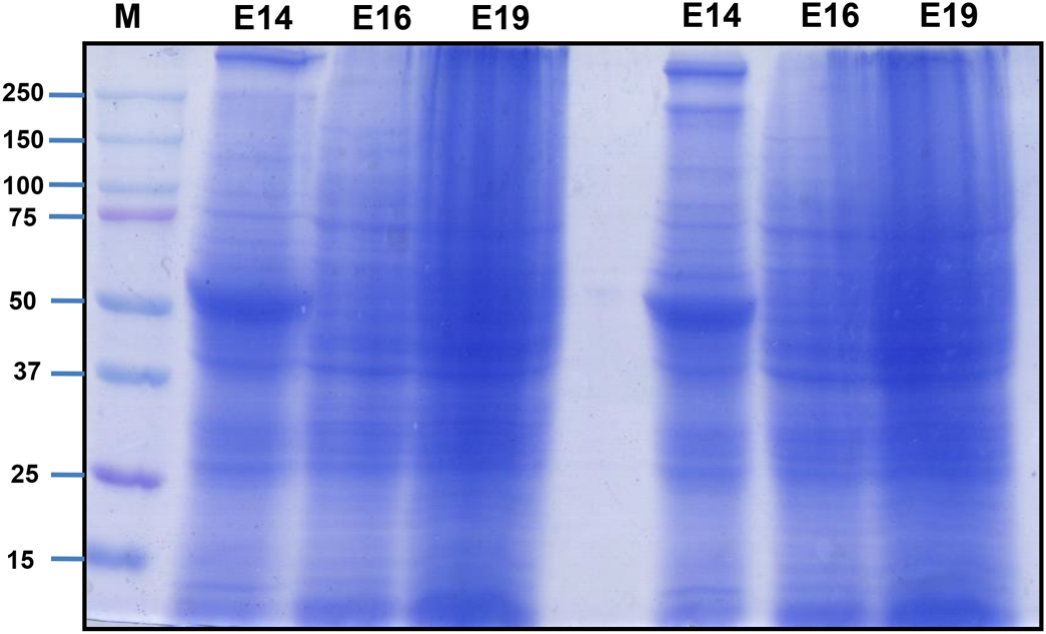
**Embryonic protein fractions of day E14, E16, and E19 were subjected to 10% SDS-PAGE and stained with Coomassie Blue.** The first well was loaded with Bio-Rad protein standards (#1610377), and the remaining wells were loaded with equal amounts of EPF from three different days.

**Fig. 3.**
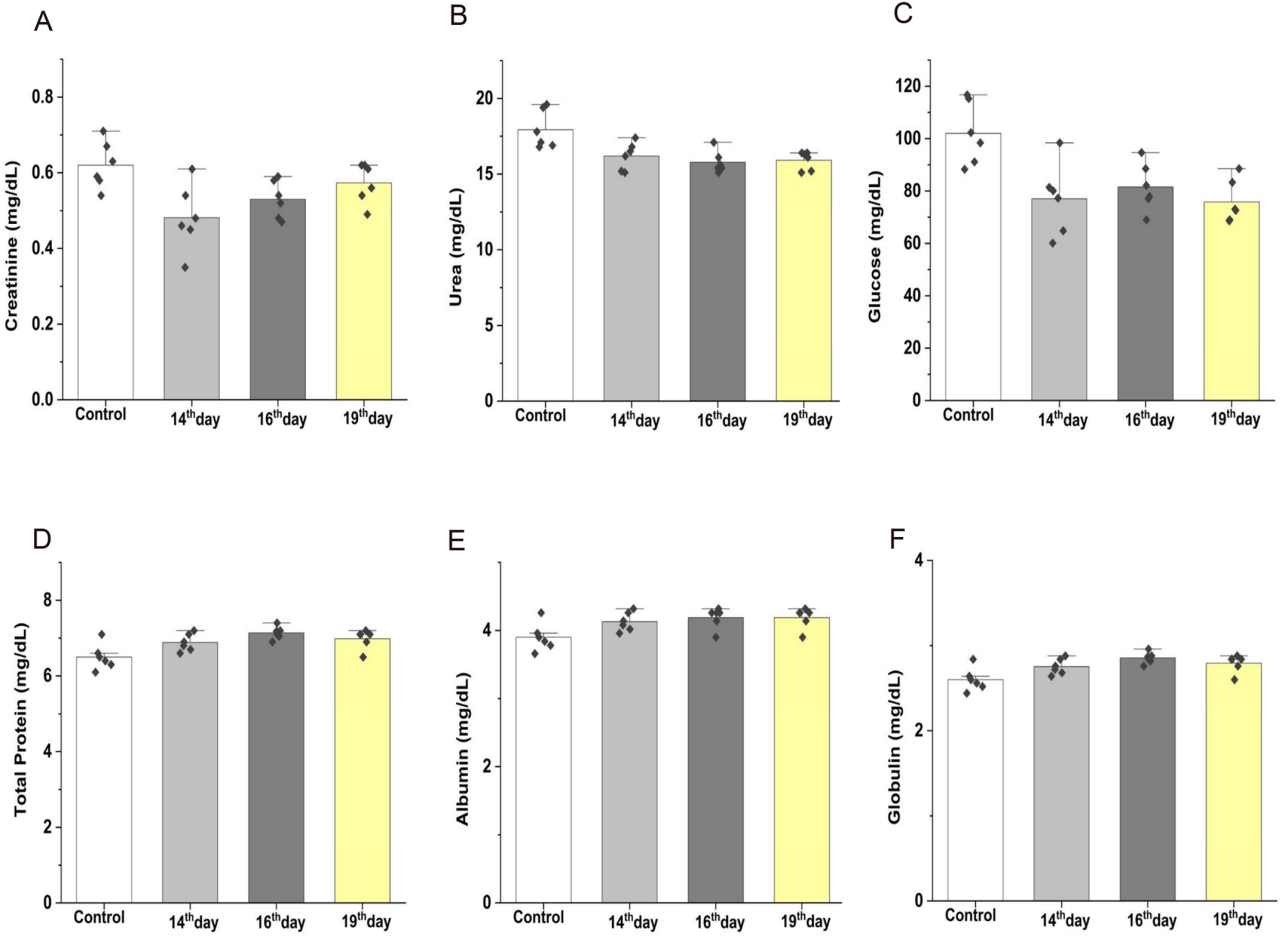
**After 48 h of each intravenous injection of EPF into 13-month-old rats, we measured (A) serum creatinine, (B) blood urea, (C) blood glucose, (D) total protein levels in serum, (E) serum albumin and (F) serum globulin levels.** Values are expressed as mean±s.e. and compared with control rats. Significance is calculated using a *t*-test where *P*<0.05.

As shown in Fig. 1, we performed inbreeding with the F1 generation of rats and isolated EPF from the mothers of F1 rats. We subjected EPF to SDS-PAGE and found that the E14, E16, and E19 protein profiles differed, with a predominant band of ∼50 kDa in the E14 extract (Fig. 2). EPF of E14, E16, and E19 from F1 mothers was injected intravenously (IV) into F2 rats with a gap of 5 days. After 48 h of each intravenous injection, we collected blood and obtained serum to estimate biochemical parameters such as glucose, creatinine, urea, serum total protein, albumin, and globulin. Similarly, we estimated biochemical parameters in serum from control rats, which were treated with EDTA. We did not notice any significant difference in biochemical parameters we analyzed between EDTA and EPF-injected rats (Fig. 3 and Table 1).

**Table 1.**
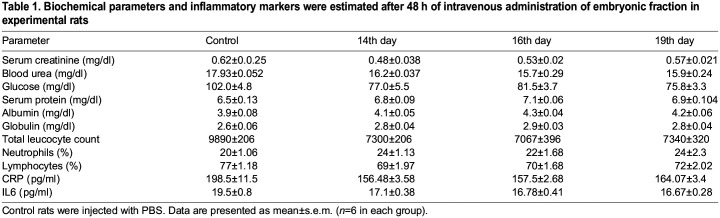
Biochemical parameters and inflammatory markers were estimated after 48 h of intravenous administration of embryonic fraction in experimental rats

### Hematological and immunological parameters were unaltered between control and EPF-injected rats

**Fig. 4.**
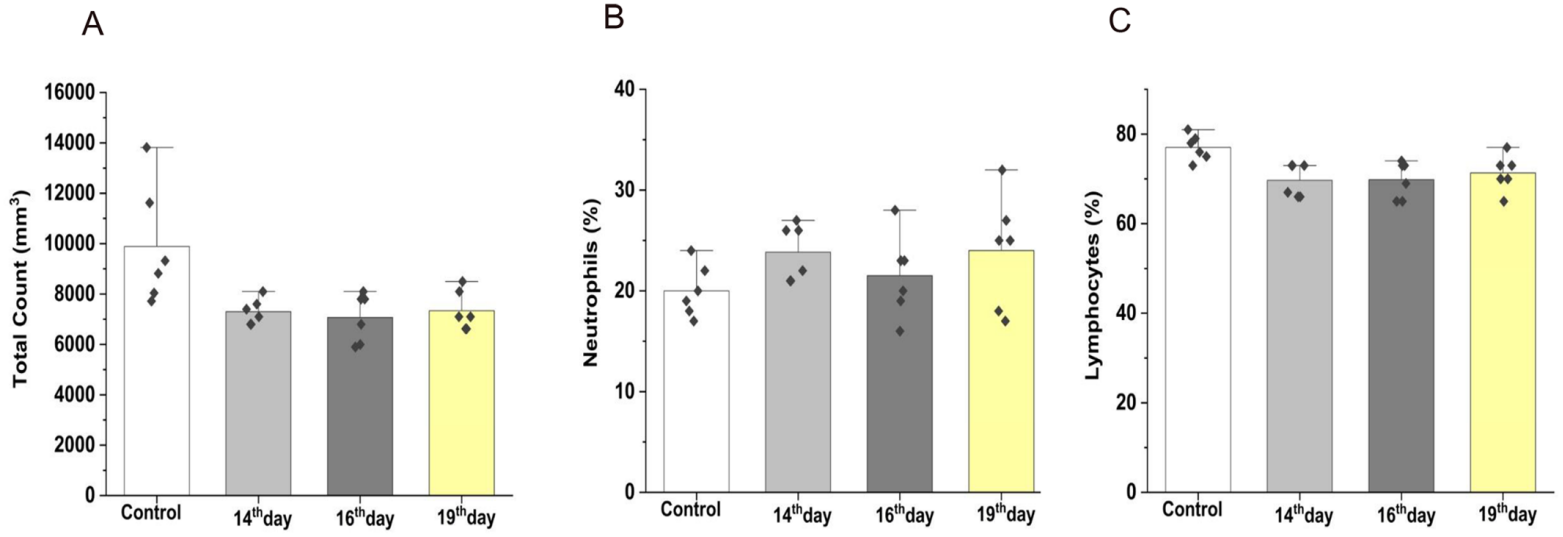
**After 48 h of each intravenous injection of EPF into 13-month-old rats, percentage of (A) total leukocyte count, (B) neutrophils, and (C) lymphocytes were measured in the blood, and values were expressed as mean±s.e.m. and compared with the control group**. Significance is calculated using a *t*-test, where *P*<0.05.

**Fig. 5.**
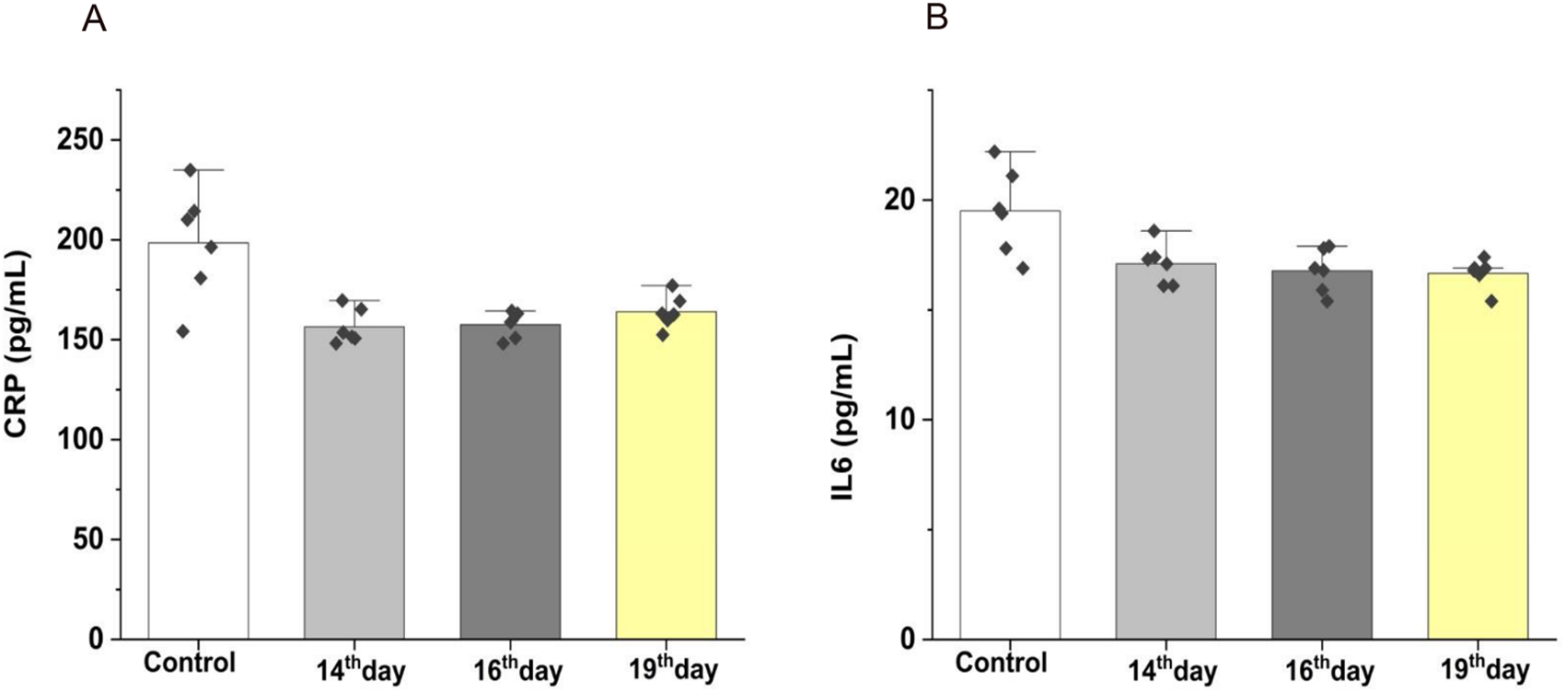
**After 48 h of intravenous injection of EPF into 13-month-old rats, we measured CRP and interleukin (IL-6) in the blood, and values were expressed as mean±s.e.m. and compared with the control group.** Significance is calculated using a *t*-test, where *P*<0.05.

Next, we assessed the impact of EPF intravenous administration on hematological parameters such as total leukocyte count and percentage of neutrophils and lymphocytes. Interestingly, EPF did not significantly alter the three hematological parameters that we assessed (Fig. 4 and Table 1). We then evaluated whether EPF could evoke the immune status of recipient rats by determining serum levels of C-reactive protein (CRP) and IL6. The immune system responds to the foreign particles by synthesizing liver-derived CRP and IL6 from macrophages. CRP and IL6 levels are comparable between EPF-administered and control rats (Fig. 5 and Table 1). These data suggest that intravenous-administered EPF did not evoke hematological and immunological aberrations in rats.

### Histological analysis of EPF-injected rats did not show pathological symptoms

**Fig. 6.**
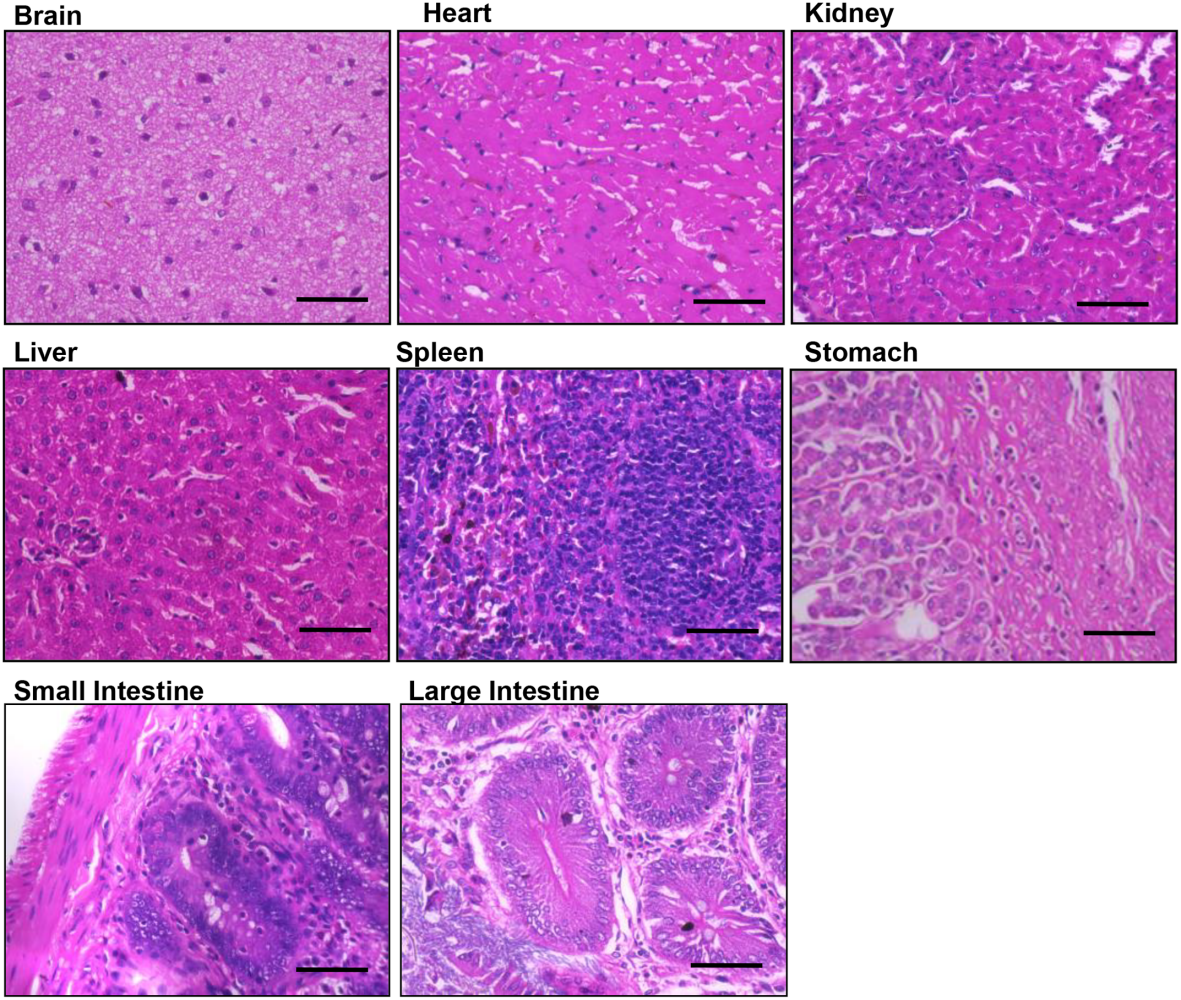
**Histochemical analysis of various tissues from EPF-treated rats were analyzed after 48 h of E19 administration.** Hematoxylin and Eosin staining was performed for the brain, heart, kidney, liver, spleen, stomach, small intestine and large intestine.

We harvested tissues from the EPF-injected rats and performed Hematoxylin and Eosin staining. Careful examination of tissues such as the brain, heart, kidney, liver, and intestine revealed no noticeable alterations in tissue architecture, teratomas, or tumors (Fig. 6). The histological data suggests that intravenously administered EPF did not elicit any adverse histological manifestations in various tissues.

### Proteomic analysis of EPF

**Fig. 7.**
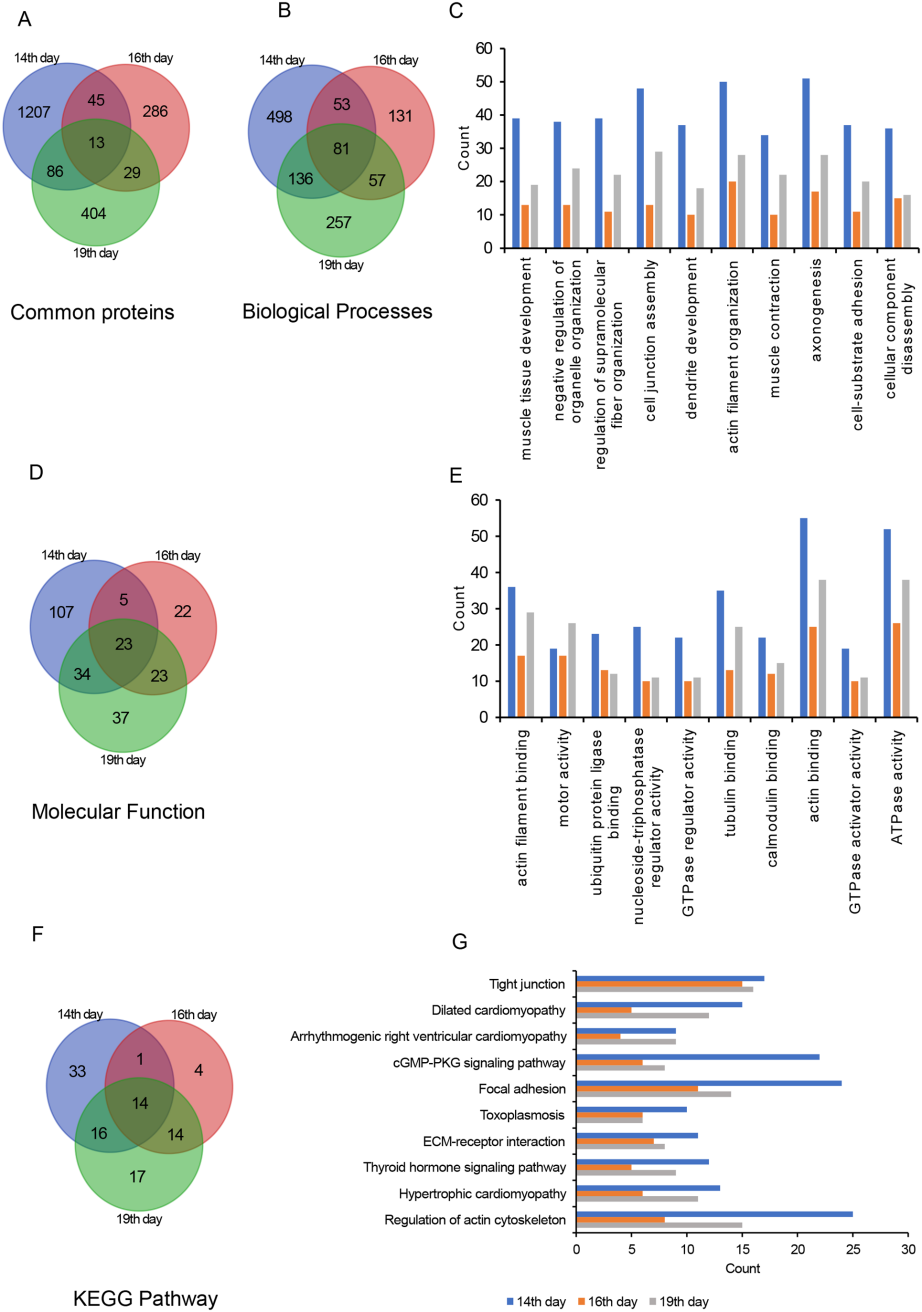
**Analysis of proteomic data.** Venn diagrams showing the number of (A) proteins common between the EPF 14th, 16th and 19th day, (B) biological processes, (D) molecular functions and (F) KEGG pathways common between the 14th, 16th and 19th day. Top ten (C) biological processes, (E) molecular functions, (G) KEGG pathways common across the EPF of 14th, 16th and 19th day. Counts represent the number of genes contributing to each process, function and pathways.

The composition of EPF was analyzed by performing LC-MS/MS analysis as described in the Materials and Methods section. The original data files were deposited in the repository (JPST003076, https://repository.jpostdb.org/) (Okuda et al., 2017). The LC-MS/MS data were analyzed using tools PLGS (Protein Lynx Global Server 3.0.2) against the *Rattus norvegicus* database. A total of 1351, 373, and 532 proteins were identified in EPF from the 14th, 16th, and 19th day, respectively (Fig. 7A and Table S1). Though some proteins were specific to the EPF of each day (E14/E16/E19), some proteins overlap between the EPF of the 14th, 16th, and 19th day (Fig. 7A). Interestingly, 13 proteins were common to all the three EPFs. Further, to understand the functions and pathways associated with the proteins identified in each EPF we performed down-stream analysis using GO and KEGG Pathway processing. Our analysis revealed some processes, functions, and pathways were unique to each EPF while some overlapped between three fractions. But, 81 biological processes (Fig. 7B), 23 molecular functions, (Fig. 7D) and 14 pathways (Fig. 7F) were common between the three sets. The common biological processes and molecular functions that were associated with proteins of EPF include muscle tissue development, dendrite development, and axonogenesis, actin binding, motor activity, ubiquitin-protein ligase binding. KEGG analysis revealed that tight junction, cardiomyopathy cGMP-PKG signaling pathway are amongst the pathways with which these proteins of EPF were associated. The top ten biological processes, molecular functions, and pathways that are common between the three time points are represented in Fig. 7C, E, and F, respectively, and are represented according to the number of genes associated with each process, function, and pathway. Since we observed a predominant band at 50 kDa in the protein fraction of EPF of the 14th day (Fig. 2), we searched for proteins whose molecular weight corresponds to ∼50 kDa and presented as Table 2.

**Table 2.**
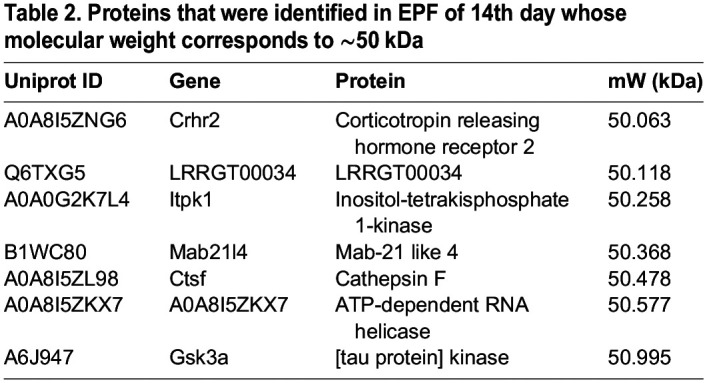
Proteins that were identified in EPF of 14th day whose molecular weight corresponds to ∼50 kDa

## DISCUSSION

In this study, we evaluated the safety of lysate consisting exclusively of proteins from rats E14, E16, and E19 by injecting them into healthy adult rats. We assessed alterations in biochemical parameters such as serum glucose, creatinine, urea, albumin, and globulin protein content. Interestingly, the biochemical parameters we evaluated after 48 h of intravenous injection of EPF were within the normal range and similar to that of control rats. Similarly, the hematological markers (total leucocyte count, % of neutrophils, and % of lymphocytes) and inflammatory markers (CRP and IL-6) did not alter as a consequence of administration of EPF. Histological analysis of most organs revealed that EPF injection did not cause any adverse effects on tissue morphology. Together, these data suggest the intravenous administration of EPF could be safe for the recipient animals at the concentrations we tested. In response to a foreign component, vertebrates react via innate and adaptive immunity. Acute phase proteins (e.g. CRP), cytokines (IL-6), neutrophils, and lymphocytes (T-cells) represent innate immunity, whereas antibodies (belonging to globulin fraction) represent adaptive immunity. When investigating the immune response to EPF injection in healthy rats, we did not observe a significant change in CRP levels, a putative acute phase protein, and no alterations in globulin fractions levels (antibodies are γ-globulins). These observations suggest that EPF did not evoke an immune response in healthy rats and could be explored further for any regenerative or therapeutic purpose.

Besides conventional therapies to deal with injured tissues or organs, there is a great advancement in the field of alternative strategies (Ramaswamy Reddy et al., 2018). Regenerative medicine is emerging biomedical research that ensures the functional restoration of tissues or organs afflicted by severe injuries or chronic diseases. The two predominant approaches in regenerative medicine that are extensively investigated are embryonic stem cells (ESC) and platelet-rich plasma (PRP). ESC, the primitive cells obtained from embryos or stem cells from the adult tissues, have the capacity of self-renewal and can differentiate into ∼200 different cell types of the adult body (Zakrzewski et al., 2019). Stem cells secrete certain growth factors and cytokines that accelerate the repair of tissue damage. Therefore, stem cells or induced pluripotent stem cells (iPSC) are explored as therapeutic options to treat degenerative or disease conditions that are presented with damaged cells, tissues, or organs. On the other hand, PRP is a component of the blood and contains five times higher concentrations of platelets (Marx, 2004). PRP nurtures those cells that can heal on their own or augment the healing process by resolving damaged tissues (Alsousou et al., 2013; Ramaswamy Reddy et al., 2018).

Apart from the above two predominant approaches, protein extract from embryonic organs is emerging as an alternative regenerative therapy. In an earlier study, Krug et al. extracted the protein fraction from the cardiac extracellular matrix of embryonic hearts and demonstrated its potential to activate endothelial cells *in vitro* (Krug et al., 1985). Rajasingh et al. reported that mouse ESC extracts promoted dedifferentiation of NIH3T3 cells, followed by stimulus-induced re-differentiation into multiple lineage cell types. Cell-free extract from mESC induced reactivation of ESC-specific transcripts in NIH3T3 cells in addition to CpG demethylation of Oct4 promoter, hyperacetylation of histones 3 and 4, and decreased lysine 9 (K-9) dimethylation of histone 3. It is noteworthy that in mouse models of surgically induced hindlimb ischemia or acute myocardial infarction transplantation of reprogrammed NIH3T3 cells significantly improved post-injury physiological functions and showed anatomic evidence of engraftment and transdifferentiation into skeletal muscle, endothelial cell, and cardiomyocytes (Rajasingh et al., 2008). Similarly, in another study, embryonic germ cell (EGC) extract showed improved reprogramming and facilitated the establishment of a pluripotent state in somatic cells, preferably by activating DNA methylation (Hu et al., 2018). The accumulating evidence argues for the therapeutic potential of cell-free embryonic extracts (Elstein, 1993; Gupta et al., 2020; Su et al., 2023). However, the safety and acceptability of these embryonic extracts were not investigated earlier. In this context, our observations play a vital role in validating the potential of EPF of E14, E16, and E19 in animal models with tissue or organ injury or chronic disease.

Proteomic analysis of EPF from the selected three embryonic days revealed the expression of a very small number of proteins when compared with the size of the proteome of a *R. norvegicus*. According to Uniprot.org, there are over 47,000 entries for rat proteome (ID: UP000002494) (Gibbs et al., 2004). Among the samples we analyzed, E14-EPF has more protein than E16 and E19 fractions. To our knowledge, this is the first study to perform LC-MS/MS analysis of rat EPF. It is unknown whether the procedure we adapted to isolate EPF has any artifacts. The common top ten biological processes identified by all three EPFs include muscle development and neurogenesis (dendrite development and axonogenesis). The data we obtained in this study were partly similar to the study of Zappaterra et al., who performed proteomic analysis of rat cerebrospinal fluid from three different time points E12.5, E14.5, and E17.5 (Zappaterra et al., 2007). Our data reveal that the expression of genes corresponding to the proteins pertinent to enriched pathways or biological processes is very dynamic. Though the expression of most genes in 14th is higher than E16 and E19, the lowest expression is in E16. The reason for the oscillation in the expression of these genes needs to be investigated.

Though we did not notice activation of innate immune response and adverse histological changes against intravenous administration of EPF, the function of various organs needs to be evaluated after extended follow-up. We have yet to demonstrate the regenerative efficacy of EPF as a whole or its components in tissue injury models. Since EPF showed no harmful effects on the overall health of the recipient rats, we are pursuing intervention studies where we induce renal injury and assess the potential of EPF to prevent or improve kidney function. In conclusion, EPF did not show any adverse effects upon *in vivo* intravenous administration in adult animals, and EPF-based therapeutic options need to be investigated in appropriate animal models. If EPF could show regenerative potential, it could at least partially substitute iPSC or ESC. Understanding the specific enrichment fractions suitable for regenerative therapy is also essential.

## MATERIALS AND METHODS

### Experiment with rats

In this study, we employed SD rats housed in an isothermal environment with a 12:12 h light/dark cycle and provided unrestricted access to food and water. The animal experiment scheme was presented in Fig. 1. Initially, we took one female and one male rat of 8 months of age and allowed them to mate. In the F1 generation, we got five female and six male rats, and these were then inbred. Mating of F1 generation rats was confirmed by vaginal swab microscopy by detecting the sperm, so that the exact embryonic days of conceived females were confirmed. Among the five F1 female pregnant rats, three rats were euthanized on the embryonic days: E14, E16, and E19, respectively. From each of the pregnant rats, ten embryos were collected and triturated to collect total embryonic proteins. The remaining two F1 pregnant females were allowed to pass complete gestation for delivery, and F2 offsprings were grown up to 13 months. F2 rats were grouped into two; controls and treatments (*n*=6 each) and among these F2 rats, six rats (controls) were injected with saline, whereas six rats (experimental) were injected intravenously with E14, E16, and E19 day total embryonic proteins in a serial manner with a gap of 3 days. Animals were purchased from Jeeva Life Sciences and the entire study was approved by the Institutional Ethics Committee of Jeeva Life Science (CPCSEA/IAEC/JLS/13/08/20/04).

### Isolation of embryonic proteins and injection into rats

The EPF was prepared as reported earlier (Goedbloed, 1980). A single embryo from each day was taken, washed five times with PBS, and homogenized using an ultrasonicator probe in EDTA (0.85 mg/ml) to inhibit the activity of proteolytic enzymes. This fraction was injected into six rats aged 13 months. Another six rats were administered with EDTA and were considered as control animals. An amount of 725 μg/14th day, 1550 μg/16th day, and 2530 μg/19th day was injected into healthy rats to investigate the adverse effects of EPF, if any. To analyze the predominant species of proteins present in EPF, we performed 10% SDS-PAGE with equal amounts of protein and stained with Coomassie Blue.

### Assessment of hematological and immunological parameters

After 48 h of intravenous injection blood was collected from rats administered with saline or EPF. We estimated blood urea, serum creatinine, serum proteins, albumin, globulin, CRP, IL-6, total leucocyte count, % of neutrophils, and lymphocytes. Biochemical parameters we analyzed using fully automated Erba EM 200 (Erba Diagnostics, Germany).

### Histological examination of tissues

Histological examination of the procedures was described previously (Adeyemi and Akanji, 2012). Organs were collected after euthanization of a single treated rat among six treatment group, as per the Committee for the Purpose of Control and Supervision of Experiments on Animals (CPCSEA) guidelines, the brain, heart, kidney, liver, small intestine and large intestine were collected and fixed in 10% (v/v) formaldehyde, dehydrated through ascending grades of ethanol (70%, 90%, and 95%, v/v), cleaned in xylene, and embedded in the paraffin wax (melting point 56°C) and stained with Hematoxylin and Eosin. The photomicrographs were captured at 100X using an Olympus microscope (CH20BIMF 200).

### Proteomic analysis of EPF

EPF fractions that were prepared as described earlier were subjected to proteomic analysis in triplicates for proteomic analysis. Trypsin digestion of EPF was performed as described earlier (Cao et al., 2019). Briefly, EPF was resuspended in lysis buffer (7 M urea, 2 M thiourea, 4% CHAPS, 1 mM DTT, and 1 mM PMSF) and the concentration of protein was measured using Bradford assay and subsequently incubated in 10 mM DTT at 56°C for 1 h. After cooling down to room temperature, the protein sample was alkylated by incubating it in 40 mM iodoacetamide for 30 min in the dark. Further, we diluted the sample to eight volumes with deionized water and subjected it to trypsin digestion (1:50 ratio (trypsin/ protein: w/w). The protein sample was digested by incubating at 37°C overnight.

Proteomic analysis of EPF was performed at Fountomics Life Sciences, Hyderabad, India (www.fountomics.com). Parameters used for LC-MS/MS were provided as Table S2 and an analysis of trypsin digested peptides was performed as detailed earlier (Cao et al., 2019). Briefly, trypsin digested peptides were resuspended in 5% ACN and 0.1% formic acid and loaded into a liquid chromatography instrument (Waters^®^ Xevo^®^ G2-XS QTof) with a BEH C18 column 75 μmx150 cmx1.7um). Mass-spectrometric analysis was performed as detailed previously (Cox et al., 2011).

Downstream analysis of the proteome was performed to check the biological processes, molecular functions, and pathways expressed on the 14th day, 16th day, and 19th day. Accession IDs of proteins expressed on each day were converted to their gene symbols using the gene ID conversion tool of the DAVID Database (https://david.ncifcrf.gov/home.jsp). ‘ClusterProfiler’ package from R studio software was used for performing gene ontological (GO) and pathway enrichment analysis. Venn diagram tool from Bioinformatics & Evolutionary Genomics (https://bioinformatics.psb.ugent.be/webtools/Venn/) was utilized for finding and representing BP, MF, and pathways respectively common between 14th day, 16th day, and 19th day proteome.

### Statistical analysis

Bar overlap diagrams were generated using Origin software. The *P*-value was calculated by *t*-test: two-sample Assuming Unequal Variances using Microsoft Excel indicated. All experiments (except the ones involving the animal models) were done at least with duplicates for each condition within the experiments. The data are represented as mean±s.e., *P*-value is less than 0.05.

## Supplementary Material

10.1242/biolopen.060266_sup1 Supplementary information
