# Supplementary material for: Safety assessment of rat embryonic fraction for in vivo regenerative therapy
Source: Biol Open. 2024 Aug 22;13(8):bio060266. doi: 10.1242/bio.060266 (PMC11360137; doi:10.1242/bio.060266)
Supplement: Supplementary information [file biolopen-13-060266-s1.pdf]

Table S1. Proteins identified in EPF from 14th, 16th, and 19th day

EPF 14th day	EPF 16th day	EPF 19th day
A6KPT5	A0A0A0MP82	A0A1K0FUH3
D3ZK88	A0A8I6AWV0	A0A0G2JSW3
A6IGL3	A0A0G2K3K2	P02091
Q6QI42	A0A8L2R402	Q63011
A6IKN9	P68370	A0A8L2R402
A6HY40	A0A8I6A3U6	A0A0G2K3K2
A6I7C5	A0A1K0GY47	A0A8I6ALV8
A6J6Q3	A0A0G2JSW3	A6I7C5
A6J5B7	P02091	A0A8I6A3U6
A6KAV6	Q6AY56	Q5XIF6
A6HHI2	G3V8R3	A6IAN2
A6HTQ6	A0A8L2RA69	D3ZRN3
A6HGC4	A0A8I6A9H1	M0R660
A6K356	A0A0G2JV65	A0A8I5ZS24
A6HGE5	A0A8I6A5N6	A0A8L2QY15
A6HDA4	A6KBM6	A0A9K3Y8C3
A0A8I6ALR3	A0A8I5Y8E3	G3V8R3
A6IRN9	P34058	A0A8I5ZT72
A0A8I6GMB4	A6KKF8	Q3KRE8
A6I7B6	A0A8L2QD97	P69897
P11517	D3ZRN3	A0A8I6AFA2
A0A8I5ZXF1	A6IKE7	M0RCB1
G3V8R3	A0A8I6G5G7	G3V7C6
Q5I0P8	A0A8I5ZKP0	A6HAQ9
A0A8I6G430	A0A8I6A5G2	A0A8I5ZR71
A0A8I6ACI2	A0A0G2JT84	E9PSN4
A0A8I6B2H5	O88753	A0A8I5ZVS3
A0A0G2K937	E9PSN4	A0A0G2K793
A6IBR7	A6H9J7	A0A8I6GMP3
A0A0H2UHA0	D3ZKG7	D3ZA84
A6HB91	G3V8Q4	F1LT30
A0A8I6A7I6	A6KND6	P55063
A6ING1	A6KHU9	A6IJ33
A0A8I6A9S2	A0A8I5Y5Y1	F1LYK3
A0A0A0MP82	F1MAF8	A0A8I6A4U2
A6JTA2	A0A8I6ABW4	D3ZE09
A0A8I5ZLC5	F7FG44	D4AB33
Q63024	Q9JLT0	A6KAI9
A0A8I5Y0U7	A0A8I6G4U4	P54258
A6JWF7	A0A0C5AMK0	A0A8I6GKP8
A0A8I6A758	B0BN43	A0A8I6APV8
A6I0V7	A0A8I6A1H4	P82995
A6HSQ9	A6JM43	A0A8I6ATU8
A6K724	P85845	A6IZR5

A0A8I5ZU06	A0A0G2KAV5	A6K529
A0A8I5ZK41	A0A0G2KB30	F1M8J7
A0A8I5ZU03	A0A0G2K162	A0A8I6G4D0
A6KUL3	A6K297	Q6B437
Q62660	A0A8I5ZQL8	A0A8I5ZYT7
A0A8I5ZWE6	A0A8I5ZXT6	A6HA77
A0A8I6ASS9	A6KU06	P14659
A0A8I6GBU6	A6KTK6	A6JLQ5
A6KHK1	A0A0G2QC02	A6HG96
M0R9X6	A0A0G2K4I4	A0A0G2K6P2
A6ING0	A0A8J8XR86	A6KGY1
A6J1V2	F1LT30	A6K8M6
F7F730	A0A8I6AFH7	A6J843
A0A8I5ZS24	A0A8I6AQV1	M0R7G2
A0A8I5ZLN9	A0A8I6AE35	A6JCG9
A6I997	A6IEZ1	F7F213
A0A8I6AGF1	A0A8I5ZTV4	A0A8I5ZP28
A6JI53	A0A8I6AFH9	Q2TUL8
M0R6R9	F1M0Y2	A0A8I5ZTJ6
B0BN24	A0A8I6ABL3	A0A0G2JSS9
A6HT35	Q8VHZ8	A6J1Z0
Q3B8R3	A0A096MJY8	A0A0G2K454
A0A8I5ZYN3	A0A8J8XLC6	A6HX51
A6HBJ5	D4A017	A0A0G2JZP4
A0A0G2KAD3	P14659	A0A8I6A566
P08723	A0A8I6GEB1	F1LVM0
A0A0G2K176	D3ZKL3	A6JDR6
M0R7G3	A0A3G1T2C5	Q4JFW1
A0A8I5ZR71	A0A096MK99	A0A8I6A8N5
A1EA77	A0A8I5ZYD4	A0A0G2JY53
O70453	O88340	A0A8I6AIN9
A0A8I5ZWF9	D4ABP2	Q6QI57
Q6R518	A6IDA0	A6IUN1
A6I9C3	A0A8I5ZM80	M0R5C9
A6IEG2	A0A8I5ZQP6	Q6TXG3
A6IEN5	A0A0G2K9R9	Q9WU61
D4AEJ6	A0A0G2JYS1	A0A8I5ZRY8
A0A8I6AFJ9	A0A8L2Q3F2	A0A0G2K2R0
A6KL33	A0A8I5ZTC7	A0A0G2JVM2
F1M834	A0A8I6AKA6	A0A0G2JYV2
Q32PX1	A0A8I5XW09	Q6TUD6
A0A0G2JTG5	A0A0G2K9A7	D3ZE12
A6K5N5	A0A8I5Y248	A6KG07
A0A8I5ZT69	A0A8I6ASQ4	A6HFH7
A0A8I6A4J9	A0A8I5YBH3	A6I7Z5
Q5J3F7	A6IGB9	A0A8I6A224

Biology Open • Supplementary information

A0A8I6GC70	A6JX22	A0A0G2K847
A6IMB2	A6J315	A0A8I5ZR04
A6JUJ0	A0A8I6A141	A0A8I5ZLN0
A6K668	A6K276	A0A8I5ZJJ6
A6JM95	A0A0G2JXJ6	A0A0G2K5P5
A0A8I6AAS3	A0A8I6ATN1	A6HCN1
A0A8I6AG02	A0A8I6G7W8	A0A0G2K7J3
A6HKS8	A0A8I5ZVU6	A0A8I6A1R7
D3ZNG2	Q9QYF3	A0A0G2K5H7
Q63360	A0A0G2JVL8	A0A0G2K3L9
A6KQB2	A6ICU8	A0A8I5ZZX9
F1LRB5	A6KFI3	A0A8L2Q3C3
A0A8I6AFR5	A0A8I5ZY79	Q9Z2B7
A0A8I6A0M0	D4A8C8	A0A8I6A1N7
A0A8I6AEI4	Q62901	A0A8I5YBU1
A0A8I5Y912	A0A0U1RS36	A0A8I6GD20
D3ZQF4	A0A8I6A6I5	Q5PPJ6
A0A8I5ZPM4	D3ZZL9	A0A8I6AGF1
A0A8L2Q170	Q6IMK4	A6I8E5
A0A0G2JVK2	B6RK61	A0A0G2K051
A0A8I6G5R7	A6JER2	A0A8I5ZXX1
A0A8I6AP59	A6IVJ1	A0A8I6AIH5
A6J8T5	A0A8I5YBN6	Q6QI37
A6IJD7	A0A8I6A6Y7	A0A9K3Y786
A6HNW5	A6K569	A0A8I6AM82
A0A8I6A6W5	A0A0U1RRY2	A0A0G2JVD2
A0A0G2JYL2	A0A0G2JTU1	A6HTR4
F1LM19	A0A1B0GWV7	A0A8I5ZJL1
A6HDI2	A0A0G2KAK2	A0A0G2K0C0
F1LWD8	A0A0G2JV16	A0A8I5ZN78
Q5RJT3	Q7TP99	D3Z9C0
A6HKK8	F1LMA9	A0A8I5ZQE0
A0A4D6YVV8	A0A0G2JZW6	D3Z810
A0A2P1NRY8	A0A8L2QDL7	A6JLQ1
A0A2P1NRX6	A6HFR2	F1M8K0
A6HC62	A6J4H7	A0A0G2JUF8
A6ISP1	A6J5E6	A0A140TAJ5
A0A8I5Y6E7	B2GUW9	Q6TXJ1
Q6MG30	Q8VI43	A0A8I6GAL6
A6I307	Q498T9	A0A8I6A1J7
A0A8I6GL71	A6HN86	A0A0H4SMZ8
A6JF12	M0RBJ7	A0A8I5Y832
A0A0G2KB11	A0A8I6AQA1	A0A0G2K481
P29348	D3ZD79	A0A0G2K344
A6IX37	A0A8I5ZQY7	A6IQV1
B5DFN5	A0A8I6GJ08	A0A8I5ZNN8

Biology Open • Supplementary information

A0A8I5ZZV0	A0A8I5ZNR1	D3ZEW7
F7ESG9	A0A8I5ZY46	A0A8I5YCF2
A6IQL2	A0A8I6G5L4	Q9ERC5
A6KFB0	A6KF62	A0A0G2KA88
Q6MG31	A0A0G2KAM8	F1LYE2
A0A8I5ZMF5	A0A0U1RRW1	A0A0H2UI26
A6KUK6	A6I6C4	A0A8I5ZPW7
A0A2P1NRY2	A0A8I5ZK69	F1M3B3
A6KAD0	A0A8I5ZT41	A0A0G2K3A7
M0RDH0	A0A8I6ABS7	A0A8I5ZM37
A6IYD8	D3ZC99	A0A8I5ZS80
A6IKE9	A0A8I6AJT8	D4A1F2
A0A0G2JTV6	A6HYC5	A0A8L2Q7U3
A6IMX1	A0A8L2UKH4	A0A8I6G498
A0A8I6A7X4	A0A8I5ZPI5	A6IUC9
Q861Q1	A0A096P6L4	Q7TQ81
Q5BJW9	A0A8I5ZPN7	A0A8I5ZR58
A0A8I5ZLL8	A0A8I6AKB3	A0A8I5ZWX3
A0A0G2K2V1	A6HUT8	A6K7G0
A6KBU6	A6JQM3	A5HK05
A6JKQ4	A6KT56	A0A8I5ZTF7
A0A0G2KBB2	A0A8I6A7B8	A0A0G2K261
F1LTC6	A0A0G2K6X6	A6HBY8
A6HSC8	A0A8I5ZM19	A0A8L2Q4D5
A0A8I6AI62	A0A0G2JV32	D3ZUE1
A6IX55	A0A8I6A8D3	A6JAQ5
A0A8I5ZP05	A6KIN1	A0A8I5ZTS7
A6HDX8	P08650	A6I924
A0A8J8XR86	F7F0E5	A0A0G2KA12
A6KCU1	A6KCL9	A0A0G2K0H9
A0A8I5Y5W5	A0A8I6G1V0	A6I1B7
A6IC87	D3ZG97	A0A8I5ZM97
A0A8I6AL01	A0A8I6GEI7	A0A8I6ASK8
A6HZB0	A0A8I6AWT4	A6ILS7
A0A9K3Y7I0	A0A8I5ZL57	A0A0H2UI30
A0A8I6A0T8	A0A8L2QH74	F1LWX6
A6HHL1	A0A8I6AL58	A6J3P5
A0A8I6A742	A0A0G2K0V1	Q29RW1
A6KIP8	A6HH26	A6ISC6
A0A8I5ZU29	A6KNE1	A0A8I6AFE5
A6JQX5	A0A8I5Y0A6	A6JNX0
F1LQL4	O08961	A6JF18
A0A0G2JTP2	P37199	A6JF92
A0A8I5ZQJ5	A0A8I5ZRQ1	A0A0G2K754
A6J2K6	A0A8I5ZSY4	A0A0G2KAF8
B1WBQ8	A0A0G2K481	A0A0G2K4R8

Biology Open • Supplementary information

A0A8I5ZUR6	A0A8I6A5C9	A0A0G2K1K5
A0A8I6AD76	Q9ERC1	A0A8I6A006
A0A8I5ZNT0	B1A2U8	D3ZL22
O08654	A0A8I5Y641	F1M8F6
A6IV79	A0A8I5ZTP3	A0A8I5Y7L4
A0A0G2KAC5	D3ZDX5	D4A5D4
A6HLZ4	A0A8I5ZRJ6	A0A8I5Y4G7
A0A8L2Q2C2	F1M090	A0A0G2K2Y9
A6J5T5	A0A0G2K022	D3ZKC8
A0A8I5YBF4	B1WCA0	Q6TUG2
F1M8D4	A0A8L2Q7A9	A6K8B6
A6KMI0	F1M0R9	A0A8I5ZZ60
A0A8I6GKK3	A0A8I5ZKZ2	B5DFK9
A6K7N9	A0A8I6ACF0	A0A8I6AA35
A0A0G2QC49	A0A8I6B5Y3	A6K938
A6IX52	A0A8I6GIU8	A0A0G2K6S9
A6I3V6	A0A8I5Y8H6	A0A0G2K3B7
B1WC12	A6HAR2	A0A8I5ZXE6
A0A8I5ZSC1	A6J6L2	A0A8I5ZV58
A0A8I5ZNG6	A0A8I5ZKQ2	A0A0G2K9S4
Q6TXG5	A0A0G2KB00	A0A8I6GJQ5
A0A0G2K7L4	A0A8L2QBV3	A0A8I6AMK5
B1WC80	Q2Q0I9	A0A8I5ZXE0
A0A8I5ZL98	A6ICR4	Q9QZ48
A0A8I5ZKX7	A0A8I5ZYD0	A6I3E2
A6J947	A0A8I5ZQ25	A6IRA3
A0A8I6GLI6	A0A8I6AQA5	A6HI24
A0A8I6A479	A0A0G2K5A4	A0A8I6ANZ5
A0A8I6AQ70	A0A8I6GHM8	A0A8I6GDA8
A0A8I6A179	M0R9L0	A0A8I6A1V1
A0A1B0GWV3	A0A0G2JT53	A6I6R9
A0A0G2JXJ4	A0A096MJC8	Q8CJB9
A0A8I5ZYE9	A0A8I5ZN27	A6KKF4
D3ZC89	A6KDU5	A0A8I5ZMB5
A0A8I5ZJ87	A6J9Q9	A6J4Q8
A0A8I6G7K6	F1LZB6	A0A0G2K484
A6HGX5	A0A8I5ZLB0	A0A8I6AP19
A0A8I5ZYG2	A0A8I5Y7L4	A6I922
M0R3P0	A0A0G2JYQ3	A6KN29
A0A8I5ZRK0	D3ZCF8	A0A8I5Y6U8
A6HTR4	A0A096MJT3	A0A8I5Y1T6
A0A8I6AH12	F1LMB5	A6KN33
F7EU69	A6J8L9	A0A8I5ZY55
Q9ESE0	A0A0G2K3B1	A0A8I6ACF8
A0A0G2K1G1	A6IC56	Q6QLM7
A0A8I5Y9W4	A6IHZ0	D3ZT58

A6KKH1	F1LQB8	A0A8I5ZZF9
A0A8I5ZNK4	A6JGN8	A0A8I5ZW86
A0A8I6AGN6	A4GTP4	A0A8I5ZS50
A0A0G2K031	A6HXD6	A6KF62
A6HUN1	A6IIU7	Q63796
A0A0G2K0Z1	A6JCE7	A0A8I6GDM7
A0A8I5ZTW3	A0A8I5ZRX6	A6J131
A0A096MKH8	A0A8I6G9R3	Q6TUH9
A0A8I6ADL4	A6HRB6	A0A8I5Y7N7
A0A8I6ACN9	A6HHA2	A6KFP3
A6I4D5	D3ZPX3	A0A8L2Q2V8
Q7TQ81	A0A8I5Y795	A0A0G2K1H0
A0A8I6ACB0	A6IS67	A6JER2
D3ZSR2	A0A0H2UHG3	A0A0G2JVX9
A0A0G2K4R3	A6HVW1	A6I1B9
A0A1L1WKK3	A6J281	A0A8I6ADV2
A0A0G2K5Y1	A6KC21	A0A0G2JW03
A6HQH3	A0A0G2K1F3	A0A8I6A2R7
A0A0H2UI26	A6HJZ6	A0A8I5ZVN0
A0A8I6GF21	Q6TLK4	A6HVW1
A0A8I6ANU4	P97690	A0A0G2JZJ3
A6IN41	A6KBW1	A0A8I5ZKA2
Q63484	Q63358	M0R9L0
A0A0G2K6P5	F1M111	A0A8I6A6V5
A6J5D1	A0A2P1NRX4	A0A8I6AIJ2
A0A8I6A1N3	A6KAA7	A0A0G2K9R0
A0A0G2K700	A0A8I5ZW36	A0A8I6AQA5
Q4QR78	A0A8I6AQ70	A6IG93
A6HUS5	A6J295	A0A8I6AKW5
A6HFT8	A6KC89	A0A8I6ALB1
A6JH55	A0A0G2K5T4	A0A8I5ZMM7
A6HS41	A0A0G2JUF8	A0A8I5ZR19
F1LWY5	D4ADD4	A0A8I5ZKE8
D3ZSW4	A0A8I5ZWR0	A6K3W8
A0A8I6AJL1	A6IXG6	A0A8I6ARD6
A0A8I6A0P5	A6HJQ8	A0A8I5ZP20
A0A0G2K9M2	A0A8I5ZWR9	A0A8I6G5U9
A0A8I5ZLM3	B5DF45	A0A8I6G9L6
A6K636	A0A8I5XVY8	A6JFG0
A6JL74	A0A0G2JWJ1	P04462
A0A8I6APM1	A0A8I5Y6D0	A0A8I6ARM7
A0A8I5ZP09	A0A8I6A9F9	A0A3G1T2C5
A6I5N1	A0A8I6A0K4	A0A8I5Y5F1
A0A8I6A1A7	A6KGX9	A0A8I5ZW88
A6HXG9	A0A0G2K1H0	F1MA36
A6IBU9	A0A0G2K6G5	A0A8I6A8R7

Biology Open • Supplementary information

P70624	A0A0G2K1L0	A0A8I5ZUN7
A0A8I5XWX0	A0A8I6AQK6	A6KM63
A0A8J8YSH5	A0A8I5ZKA2	D4AC33
A0A8I6AHZ9	A0A8L2Q1J0	A6KQA8
Q5BJU9	A0A0G2JZQ9	A0A0G2JVU1
A0A8I6AL50	A0A0G2K0P3	A0A8I5ZTX2
A0A8I6AB79	Q6MFX8	A6J2V3
M0R8R4	A0A8I5YC57	A6KGV0
M0R553	A0A1W2Q6M3	A0A8I6A142
A0A2Z5ZBL4	A0A0G2KAA5	A6JNP4
F1LTB8	A0A0A0MY22	A6HHK3
Q4V8E7	A6HE24	A0A8I6ACB7
M0RDJ3	A0A0G2K7M4	A0A0G2K1L0
A0A8I6AQ25	A0A8I5ZJ94	A6I3U3
F1M4D5	A6IJ33	A0A8I6GIV4
Q5M854	A0A8I6AB90	A0A8I5ZNS1
A0A0G2JUR3	A0A8I6ARY9	A0A0G2JXU8
A0A8I5ZQ56	A0A8I6AM21	A6I5D8
D3ZS84	A6ICM2	A6JN24
A0A0G2JUF3	A6KPM4	A6IBC6
A0A8I5ZYK8	A6JVK5	Q7TP87
A0A8I6AEE0	A0A8I5ZJF2	P49744
A6K6Q9	A0A8I6AA04	D3ZNX0
A6JYT8	A0A140TAF6	A6K6G3
A0A8I5ZY55	A6I359	A0A8I5ZM44
P07314	A0A8I6ARM7	A0A0G2JTD7
Q8K3R0	A0A0G2JUJ9	A0A0G2K070
A0A8I6A4Z6	A6KJI9	W8C8J2
A6KD50	A0A8I6GE00	A0A8L2Q868
A6K2Q7	A0A8I6ABC1	A6HAK0
F1M8H8	D3ZUK4	A0A8I6AG58
Q5BJU8	A0A8I6AA75	F1M748
A0A8I6AGB5	A0A8I6A9T7	A0A8I5Y618
A0A8I6AHF8	A0A0G2JV77	A0A0G2K1Q8
A6HEF4	A0A8I6ANR4	D4AA77
A0A8I6A3T6	P70569	A0A8I6AML7
A0A0G2KAU3	P10688	A0A8I6G3Y6
D4A7B6	A0A0G2K231	A0A8I6A095
A0A140UHX4	Q9Z0Y8	A0A0G2K8N9
A6HVP6	D3ZNS6	A0A8I6A687
A0A8I5ZLM7	A6KTD3	A6KIE5
A6JLM5	A0A8I6A5J5	F1LV10
P23785	A6KUQ2	A0A8I6AKG9
A0A8I6AVR5	A6HMN7	A0A8I5ZRW7
A0A8I5Y6T4	A6JJC3	F1M6S6
A0A8I6AQN7	A0A8I5ZWT2	A0A0H2UHP5

Q6IN02	A6JSR8	A0A0G2K1A9
A6KEL1	A6I896	A0A0G2JTM7
A0A8I6A5Y8	A6HHQ4	A0A8I6AMK8
A0A8I6AS15	A6IL73	A6IX92
Q5GH56	A0A0G2JZF2	B6RK61
A0A0G2K1G4	F1LXT0	A0A0G2K3H1
A6KPI2	A0A8L2QSN0	A0A8L2Q7U5
A6J3V6	A6JQM5	P43427
F1LSL4	Q7TP01	A0A8I5ZKI0
A6I061	A0A0G2K5P5	A0A1W2Q667
A0A8I6APP6	A0A096P6M3	A0A8I5ZRT6
A6K5R0	A0A0G2K0H1	A6HGQ1
Q66HD5	A6IMM4	F1LV13
A0A0G2K7V6	D3ZT58	A0A0G2K3C9
A0A8I5ZU77	A0A8I6ALY5	A0A1W2Q629
A0A0G2K0K3	A0JPQ0	A0A8I6A5G5
Q6GX86	A0A8I5Y8C0	A0A0G2K0F5
F7EWM3	A6JSC6	A6HLX5
A6J5U1	A0A8I5ZRA1	A0A8I5ZJ95
A6ITV2	P02564	A0A0G2K162
F1LS93	A0A8I5Y739	F1M3X5
A6HIV1	A6HZC7	A0A0G2K999
A0A8I5Y5P7	A0A8I6A726	A0A8I5ZKW7
A6IYB1	A0A8I6A1N2	A0A0G2K0V1
A0A8I6A4K5	A0A8I5ZPJ4	A0A8I5ZYI6
D4A2K4	A0A8I6AB71	A0A0G2K8D8
A6ILV3	A0A8I5ZUC5	A0A0H2UI34
A0A9K3Y763	M0RB12	A0A0G2JUH8
A0A8I6ARF8	A0A0H2UHQ2	A0A8I6AGS6
A5HK05	A0A8I6AD42	A0A8I5ZKQ2
A6IE96	D3ZW82	A6J315
Q923I8	A0A8I6AI92	A0A8I6A621
Q68G29	A0A8I6G9B4	A6KT59
A6KU68	A6JFQ3	A0A8I6AR97
A0A8I6AJD2	A6HXC8	A0A8I6A1U5
A0A8I5ZQG7	A0A8I6ALZ3	A0A1B0GWS3
F7EVW7	A0A8I6GFC1	A0A8I6A4B4
D3ZAS7	Q9JHL3	A0A8I5ZNR1
A6K934	A0A0G2K3H1	D3ZH67
A0A8I6AB30	A0A0A0MY11	D4A0A1
A0A1W2Q641	A0A0G2JZX5	A0A8I5ZSB0
A0A8I6AEK4	A0A0G2JYF2	A0A8I5YC40
A0A8I6A3W5	A6J730	A6I3B6
A0A0G2JUA6	A0A0G2JZC6	A6I1F6
A0A8L2QEL3	A6JF18	Q641W3
A0A8I6A4D7	A0A0G2JV69	A0A8I6G926

Biology Open • Supplementary information

P02770	Q6MG71	A0A8L2QSN0
A0A8J8YF16	F1M5W3	A0A0G2K946
A6I5V6	A0A140TAA3	A0A8I5ZNR8
A6JBQ2	A6JVP5	P01026
A0A8I6A4G5	A0A8I5ZS43	A0A096P6M8
A0A8I5ZWQ0	A0A0G2K2Y8	A0A8I6A6L3
A0A0G2K659	A6HXE0	A0A8L2Q2G6
A0A8I6AAJ7		A0A8I6A7M4
A6JUL0		D4A4Y9
A6JCG8		A0A8I5ZT40
A0A8I6APZ0		A6JXI4
A0A0G2K950		A6IND2
A0A8I6A6A6		A0A0G2K2S5
A6I1G5		A6KGX9
A6I630		A0A0G2K6W6
A6KHA6		A0A8I6AAE2
D4ACV0		Q63541
A6HWD4		A0A8I6GIP5
A0A8I5ZRM0		A0A1W2Q6Q0
A0A8I5ZZU5		A0A8I6G5N4
A6HYP4		A0A0G2JWS1
A6JWY1		A0A0G2JY35
A0A0G2K2X2		A6HZG0
A0A0G2JYB6		A0A8I5Y585
A6J158		D4A5F5
A0A0H2UHY4		A0A0G2K3V1
A0A8I6AIQ6		A6KA00
A6HM43		A0A8I5ZNY8
A0A8I6APX7		A6J281
D4A864		F1LWV6
A0A8I6AE10		A0A0G2K952
A0A8L2QJI4		A0A0G2JVV8
A0A0G2JSV1		D3ZIT7
A6JA07		A0A0G2JYK6
A6J7J3		D3ZDX5
D4A7H1		A6J668
A0A8I6AZW3		A0A8I6AUW1
A0A8L2QDI1		A0A0G2K2N2
Q3LUD4		A6KEA5
P38659		A6KI51
O54858		A6IBA2
D3ZGJ1		A0A8I6AHW2
A0A0G2JSY2		A6KMX3
A6HR21		A0A8I6A2C8
A0A8I6G2A6		A0A0G2JUU1
A0A8I6G9N7		A6I6X2

Biology Open • Supplementary information

A0A8I5ZTC7		A0A8I6AHN4
A6HR86		Q9Z0U5
A6I8V0		A0A8I6AN44
A6I2H3		A0A8I6GL06
A0A8I6A810		D4A3N4
F1LT76		A6IGB4
Q6J0M8		A0A0G2K7B6
B5DEF7		A6HPH8
A0A0G2KAX6		A0A5H1ZRT6
A6JPF6		D4A959
A0A8I6A8Q2		B2RYA4
A0A0H2UHU8		Q6ZYE7
A6I254		D4A0U0
A0A8I6GK91		A6IC30
A6H9I7		A6HH26
A0A8I5ZT94		A0A8I5XVS2
A6HMG5		A0A8I6GAU1
A0A8I6AM15		A6ITC7
A0A8I6GDJ0		A0A8I6A240
A0A8I6ACH9		A0A8L2UH90
A0A8I5ZP81		A0A0G2JXH7
A0A8I6A3G7		A0A0G2K1E5
A0A8I5ZR58		A6KG18
A0A0G2JY81		F1LX08
A0A8I5ZKW6		D4ADD4
A6J5E1		A0A8I6A8A4
Q9QZX1		A0A0G2K860
B2RYB9		Q5EB81
D3ZWX4		D3Z9R6
A0A0G2K7C9		P55266
A0A0G2KAW9		A6IEZ2
A6HUD1		A0A8I5ZK89
A0A8I6A342		A6IZA1
A6IFX2		A0A140UHX6
A0A8I5ZLT8		A0A8I6ASN6
A0A8I5ZNT5		A0A8I6AB89
D3ZJ87		A0A8I6A7A1
A0A8I6AJP4		A0A8I5ZQI0
A0A8I6AQR4		A0A8I6AQV7
A0A1W2Q6G2		A0A8I6A9D5
A0A0G2K324		A0A0G2JY08
Q62847		A0A8I6AC46
A6J352		A0A8I6GIK4
B1H296		F1M0H0
A6I432		Q4V8M7
A6I315		A0A8L2R519

Biology Open • Supplementary information

A6IL12		A0A8I6A0J4
A0A0G2K3F3		A0A8I6AMR5
A0A8I6GF99		A0A8I6AH44
A0A8I6A6M4		A6IIT7
A6J4Z5		A0A8I6AF96
A0A0G2K628		A0A0G2K2J7
A6IUH0		A6JAG1
A6JD69		Q923Z7
A0A8I6AM74		A0A0G2K8A6
A6JMF5		A6KRR8
A0A8I5ZNU1		A0A0G2K237
A0A0G2JVG6		A0A0G2K666
A6KGJ3		P15390
A0A096P6L4		A6J2K8
A0A8I6AI85		A0A8I6GJJ5
A0A0G2JXS2		A6J208
A0A8L2RBI0		A6KM30
A6KBX5		B1WC18
A6IDC6		Q6AXP9
A0A8I5ZUI2		D3ZV81
A6IIM4		A0A8I5ZQ48
A0A8I6A1K0		A0A8I6A6A9
A6KG48		A0A8I6AT16
A0A8I6AKX9		A0A8I6A160
Q63603		A0A8I6AHV3
A0A8I5ZPG6		A0A8I6AVM6
A6KMF3		A0A8I6AL58
A0A8I5ZRP8		A0A8I5ZQH5
A6JDM3		P24464
A0A8I6AMR1		F1M048
A0A8I6A0J4		A0A0G2JVH8
F1M453		A0A1B0GWZ0
Q7TMB7		A0A0U1RRS6
F1M5P6		A6J7M5
Q9R1D1		B2RYB1
D4A4Q1		A0A8I6A8B2
A0A8I6A6X9		A0A0G2K8L7
A0A0G2JVU1		A0A0G2JYM3
A6I5Y1		A6JXR9
A0A0H2UHW9		P97603
M0RDD8		A0A8I5ZWC0
A0A8I6AKM5		A0A0G2K2Y2
A0A8I6API0		A0A8L2QDI1
A0A8I5ZXZ7		Q6QI92
A0A8I5ZR07		A6HPF6
A0A0G2K0S6		F1M9Q3

Biology Open • Supplementary information

P30835		A0A8I6AAA8
A0A0G2KAA9		A0A8I6ADH1
A6J351		A0A096MJS9
Q3SWT4		A0A8I6GK80
A0A8I6A4E3		A6IEZ1
F1LWE4		A0A0G2K4H7
A0A0G2K5U5		Q7TP94
A0A8I6GCJ9		A0A8I6A019
A6JZ60		A0A0G2K8Z9
P70505		M0R7L1
A0A096MJ75		Q2I7M7
A0A8I6AQ43		A6III1
P21463		A0A8I6AS76
A0A8I6AH58		A6JND0
A6HRV7		A0A8I5Y2A3
A0A8L2QEB4		A6HJX8
A0A8I6GGP5		A6KSQ2
A0A8L2UQK5		A0A8I6AGI3
A0A8I6A4S8		A0A8I5XWN5
A0A0G2K0I6		A0A096MJY0
A0A8I6GDF6		A6KS37
Q6TXG3		A0A0G2JTB0
A0A0G2JUJ9		A6KGK2
A6HZ97		A6KHY4
A0A8I6AFX4		A6I191
A6KF55		A0A8I6A974
D3ZAH0		A0A8I5ZZ86
A0A8I5ZMU9		D4AB99
A6ISZ7		
A6ITW0		
A6JST1		
F1LW07		
A6HZ98		
A6I262		
A6J1T0		
O70368		
A6J1E4		
F1LWF2		
A6I0X9		
A0A8I5ZM57		
A0A0G2K0Q8		
A6KR61		
A0A8I6AMT6		
A0A8I6AC09		
A0A8I6AMQ9		
A6JFW3		

Biology Open • Supplementary information

A6ISW5		
F1LT30		
A0A8I6AL23		
A0A8I6A9L8		
A6JHH1		
D3ZJM7		
A0A0G2JVY4		
A6J705		
A6HWU4		
A0A096MKA9		
G3V887		
A6I4B4		
A0A0G2JWL2		
A0A0G2JZL7		
A0A0G2K607		
A0A8I6A7I7		
M0R6D9		
Q9QX72		
A0A8I5ZYG1		
A0A0G2K5X6		
D3ZB52		
A6IGT8		
A0A8I5ZZS9		
A0A0G2K2X9		
A0A8I5XWF1		
A6HUX6		
A0A8I6A486		
A0A8J8Y893		
A6K6R2		
D3Z9H7		
A0A8I6G230		
A0A8K0ZPU7		
A0A0G2K685		
A0A8I6GH61		
D3ZCT5		
A0A8I6AFB4		
A0A0A0MXT6		
Q03351		
A0A8I5ZKK0		
A0A0G2K2H0		
A0A8I5ZU94		
A6HZ88		
A6IRV5		
A0A0G2JZ86		
A0A0G2JTR4		
A6KQP6		

Biology Open • Supplementary information

A0A8I6GJR4		
D3ZJF1		
A0A8I6GLR2		
A6KJL4		
A0A8I5XWQ1		
D3ZME2		
F1LZV3		
A0A8I6AC88		
D3ZFX7		
A0A0G2JTB3		
A6HCV0		
D4ACG6		
A0A8I5ZQW6		
A6I183		
A0A8I5ZKZ0		
D4AAT1		
A0A0G2K9J6		
E0YJE6		
A0A8I6GEV8		
O35803		
A0A8I6AM26		
A6ILB5		
A0A0G2KA90		
D3ZEQ5		
A0A8I6A145		
M0RAR2		
A0A8I5ZYW5		
A0A8I6AMQ0		
A0A8I5ZWM9		
A0A8I6A1T2		
A0A8I5Y236		
A0A0G2JXN8		
A0A8I6AIR1		
A6KCF0		
A6I0J8		
A0A8I5ZPC2		
M0R3S3		
Q767I9		
A6HV14		
A6JZB2		
A6JVP8		
A0A8I6A9Q2		
Q6TXF0		
A0A0G2K783		
F1MAL4		
A0A8I5ZM90		

Biology Open • Supplementary information

A6IZK4		
A0A8I6A680		
Q593X7		
A0A0G2JU76		
A0A0G2K8D8		
A0A8I6G9B5		
A6HJQ8		
A0A8I5Y6Q3		
A0A0G2JY62		
A0A0G2JT84		
A0A8I6A6C6		
A0A8I6A472		
Q63767		
A6KIK6		
A0A0G2JTA7		
A0A1B0GWU1		
Q767H8		
A6JLU9		
A0A8I6AA99		
A0A8I5Y6Z0		
A0A140TAC8		
F8WFW5		
D3ZAH4		
O88761		
D4A8C8		
A6HUT8		
A0A0G2K7P2		
A0A8I6GM92		
B2RYB1		
A0A8I6A624		
F1M0J7		
A6KF82		
A6KPJ9		
D3ZJ92		
A0A8I5ZVC4		
D3ZU09		
A6JQK2		
A0A8I5XW81		
A0A8I5ZLW4		
A0A8I5ZLP6		
A0A0G2K4H7		
A0A8I6B2I8		
A6HPX1		
A0A8I6AC41		
A0A8I6B1X1		
A0A8I6A812		

Biology Open • Supplementary information

Q6QI35		
F1M4B6		
A0A1W2Q6N4		
B2RYL7		
A0A8I5ZUH8		
A6J668		
A6I6N1		
A0A8I5ZZK0		
A0A8I6AD87		
A0A0U1RS37		
A0A140TAG3		
A0A0G2KBC4		
A0A8I6ABS6		
A0A8I6ALB6		
A0A0G2K261		
B5DFJ4		
A0A8I5ZY72		
A0A8I5Y285		
A0A9K3Y6U0		
A6KPG3		
Q7TPL1		
A0A8I6AQL5		
P54757		
D3ZLP9		
A0A8I5Y1Z8		
M0R4R2		
A0A8I6APK9		
A0A8I5ZJA8		
A0A8I5ZVI3		
Q8K4K5		
A0A8I6AN95		
D3ZD72		
Q4FZU4		
A0A8I6G9F8		
D3ZP31		
A0A8I5ZRD0		
A0A0G2JVH5		
A0A0G2K8Z6		
F1M280		
Q9Z221		
P69526		
A0A0G2JUZ5		
A0A8I5ZQ75		
Q6W3B0		
A6JXR9		
A0A0G2K4Z7		

Biology Open • Supplementary information

A6IXL9		
A0A0G2K9I6		
A6HFL1		
Q10728		
Q5YLM1		
D3ZCC2		
A0A096MJF9		
Q7TMA9		
A0A0G2JX92		
A0A8L2QHF2		
Q5RKH1		
Q9ERE6		
Q6XDB6		
A0A8I6A8E8		
A0A8I6A138		
A0A8I6A860		
A6HQV2		
A0A8I6ASR2		
A0A8I6AED4		
P97837		
A0A0G2KAI2		
D4A318		
A0A8I5Y8E5		
A0A8I5ZRK1		
A6KH53		
A6KIQ1		
A0A0G2JZC4		
A6J4H7		
F1LSD5		
A0A0G2K033		
A0A8I5ZTX4		
A6HGU9		
D3ZTD8		
Q5VJM4		
A0A0G2K6J1		
D3ZYY9		
A0A8I6A7A3		
A0A0G2JVE4		
A6IR03		
Q9Z1X1		
A0A8I5XVC5		
G3V703		
A0A0G2JV75		
A0A8J8XTR6		
A0A8I5Y0P2		
A6IE09		

Biology Open • Supplementary information

D3ZNU5		
A0A0G2K5K1		
A0A8I6G1V0		
A0A0G2JYU6		
A6JHY5		
F1MA23		
A0A0G2K132		
A6HAQ0		
D3ZM84		
A6IMI1		
F1LPP1		
A0A0G2KAF5		
M0R6H5		
F1M5F3		
A0A8L2Q6Y5		
Q6QI57		
A0A8I6AET0		
Q62924		
P54258		
A0A0H2UHB2		
A0A8I6AFY2		
A0A8I6AD92		
Q499M6		
A6JV49		
D3ZX21		
A6IEZ2		
A6HEC4		
A0A8I5ZQV4		
A0A8I6AQV1		
A0A8I5ZZ36		
A0A0G2QC41		
F1LS35		
A0A0G2K4K4		
O08962		
M0RBE7		
A6K4J3		
A0A8I5Y118		
Q6TUH9		
A0A0G2JYK2		
A6J3F5		
A0A8I5Y7S8		
A0A8I6A871		
Q4VSI4		
F1M0R2		
A0A8I6A1N7		
A6J805		

Biology Open • Supplementary information

D3ZRM0		
A6KBW3		
A0A8L2Q989		
A0A0G2K8U2		
A0A8I6A850		
A6KHC6		
A0A8I5YCM3		
A0A0G2JV24		
Q6TUF9		
A6K8J6		
Q3ZBA0		
D3ZT56		
A0A0G2K8V1		
A0A8I5ZW37		
D3ZWI4		
A6I5T3		
A0A8I6A183		
D3ZUV0		
A0A0G2K8Q3		
A6JR89		
A0A8I5ZU24		
A0A8I6A1R1		
A0A0G2JZV3		
A0A8I6A3U3		
A6KDR4		
A0A8I6AHS6		
A0A0G2K0I0		
A6J1T2		
P47245		
A0A8I6A3Q6		
D3ZA74		
A0A0G2K0V4		
P97573		
A6HFH8		
A0A0G2K7L2		
A0A8I5YC57		
Q62976		
A0A8I5ZJB6		
A6I286		
M0R4T2		
A0A8I5ZL13		
D3ZVW3		
A0A0G2JYZ1		
A1EC95		
A0A8I5ZZQ5		
A0A8I6A294		

Biology Open • Supplementary information

Q7TMZ9		
A0A8I6A5K9		
A0A8I5ZS10		
F1LNY6		
Q9JKB5		
A0A8I6G8P4		
A0A0A0MY13		
A6IBQ0		
A0A0G2K1Y7		
A0A8I6GEF5		
A0A096MKI6		
A0A8I5ZY39		
A0A0G2K5M9		
A0A0G2K5A7		
Q6MG72		
M0R8P6		
A0A8I6A598		
D9I2G1		
A0A8I6AQ81		
A0A0G2K5W1		
A0A8I5ZN42		
Q9Z2Y1		
A0A8I5ZQY2		
Q9JLS3		
A0A8I5ZZS3		
D4A024		
A0A0G2JZ76		
A0A8I5Y6H6		
A0A8I6AU50		
A0A0G2K4K6		
A0A8I5XZV4		
A2RRU4		
A0A8I5ZPI5		
A0A096MKH1		
D3ZKE0		
A6ICN2		
A0A8I5ZR15		
A0A096MK07		
A0A8I5ZV39		
A0A8I6AUM9		
A0A8I5ZMR5		
A0A0G2K1Y9		
A0A8I5XVK6		
Q05695		
A0A8I5YBF0		
P43245		

Biology Open • Supplementary information

B5DFK9		
D3ZS39		
A0A8I6A0V7		
M0R685		
A0A8I5ZKI0		
Q6TUF6		
A0A8I6AAY6		
A0A0G2K283		
A0A8I6AM59		
A0A0G2K3H5		
F1LMS4		
A0A8I6AB09		
A6KLB2		
A6I923		
A0A8I6AS75		
D3ZT03		
A0A8I5ZYZ3		
D3ZH11		
A0A8I6A388		
A0A8I5Y093		
D3ZIZ6		
A0A8I5ZWM6		
A0A8I6A0C1		
F1LQB8		
A0A8I5ZYN4		
A0A1B0GWM7		
A0A8I6AUW1		
F1M3T8		
A0A8I5ZLK6		
A0A0G2JT63		
A0A8I6A413		
A6K9F8		
A0A8I6G6R3		
P22985		
A6H9Z6		
E9PTB6		
A0A0G2JZE4		
A6IBP3		
A0A0U1RRS6		
A0A8I6GH02		
A0A8I5ZV07		
D3ZZJ4		
D3ZL75		
A0A8L2QSS8		
A0A8I5ZTS7		
A0A8I6AF46		

A0A0G2K6E8		
D3ZT58		
A6JY17		
A6KTE8		
A0A0G2K4R8		
A6HC94		
A6JQY0		
A6K4T1		
A6KNQ6		
F1M1B9		
A0A1W2Q695		
A0A8I6AEG8		
A0A0G2JTD1		
A0A8I6GAW9		
A0A8I6A160		
A6KFG0		
A0A8I6AEP0		
F1LPC6		
A6I0M8		
A0A0G2K3C8		
A0A8I5ZR67		
D3ZE09		
A0A8I6A895		
A0A8I6GLB3		
D3ZCW3		
D3ZHW0		
A0A8I6GKX2		
A0A8I5YBX7		
A0A0G2JY58		
A6JYM5		
D3Z9F7		
A0A0G2JWH3		
A0A8I5ZMZ8		
A0A0G2JZM8		
A0A8I6AIH4		
D3ZQW1		
A0A8I5ZVL0		
A0A8I6A9Y3		
A0A0G2K7L6		
A0A8I6A6D9		
A0A8I6GKD6		
A0A8I5ZQQ6		
A0A8I6ATT8		
A6HXC3		
Q6QI76		
D4A224		

Biology Open • Supplementary information

Q9QX74		
Q6MGB2		
A0A0G2K5U0		
A0A8L2QY59		
A0A8I5ZTM3		
Q9WUI9		
A0A8I6ANN3		
A0A0G2JUX4		
A0A8I5ZW14		
A0A0G2K5A4		
A0A0G2K5V8		
Q6IE52		
Q498D4		
A0A8I6GEB1		
A0A8I6AM13		
A0A0G2K769		
A0A0G2K588		
A6JIA5		
A0A8J8YM62		
D4A4Y9		
A6I4C6		
A0A0G2KAM8		
A0A8I5ZSB0		
A6K5N8		
D3ZWJ2		
A0A0G2JZE7		
A0A0G2JWB6		
A0A8I6AVL2		
Q499R0		
Q62635		
F1LTT7		
Q8VI43		
A0A8I6G8E6		
A0A0G2K6N2		
A6HMI7		
A0A096MK13		
D3ZGP6		
A0A0G2KAU9		
D4ADF6		
A0A0G2JZW6		
A0A096MJG5		
F1LVM0		
A6IDZ3		
A0A8I5ZST1		
A0A0G2JUI5		
A6HAR2		

Biology Open • Supplementary information

A0A8I6AIB8		
A0A0G2JUF8		
A0A0G2KA49		
A6IUD6		
A6IDA0		
A0A0G2K7N9		
Q62766		
O70608		
A6J5V4		
A0A0G2K231		
C0HL12		
A0A1W2Q6D5		
A0A8L2QEC9		
A0A8I5YBQ0		
A0A8I6G721		
A6KMX3		
Q07310		
A6HVA9		
D3ZET9		
A0A0G2JYA7		
A0A0G2K3N6		
A0A0G2JYJ4		
A6HRB6		
A0A8I6AFH2		
D3ZU13		
A0A8I5ZSQ5		
A0A8I6AC12		
F1MAA7		
E9PT59		
A0A140TAF3		
A0A096P6M8		
D3ZUF0		
A0A0G2JX47		
A0A0G2K6V2		
A6HV70		
A0A0G2KA11		
A0A0G2JXJ0		
D3Z9N6		
A0A8I5ZTH3		
A0A0G2KA88		
A0A8I5YCN5		
A0A0G2K0H3		
A0A8I5ZSV0		
A0A8I5XVR7		
Q6TXJ1		
Q6DUH4		

A0A0G2JTN3		
D3ZLS6		
A0A0G2K320		
A6KGK0		
Q7TP73		
A0A8I5ZXU1		
F1M9X0		
A0A0G2K042		
A0A8I5Y0L1		
D3ZM20		
A6J7X8		
A0A0G2K5P5		
P01026		
Q6ZYE7		
A0A0G2K6T6		
A0A0G2K3S4		
A6I3V1		
A6I1L5		
A0A8I5ZUU6		
A0A8I5ZNN8		
F1LU18		
A0A8I6AIJ2		
A0A8I6AQM8		
A6J9C3		
A0A8I5ZDN9		
A0A8I5Y6D0		
F1LZ05		
P70570		
A6KEB3		
D4ACN5		
A6HR18		
A6ICR4		
A6J4Q8		
A0A8I5ZS43		
A6IUI3		
A0A8L2Q7U3		
A6IBL0		
A0A8I6A8D3		
A6IBX1		
A0A0G2K1Q2		
D3ZMD6		
A6IA93		
F1M378		
A0A8I6AN33		
A0A8I5ZYD4		
A0A8I5ZJI9		

Biology Open • Supplementary information

A0A8I5ZNG4		
A0A0G2K8R3		
P97706		
A0A8I5ZMW9		
A0A0G2K499		
A0A8I6AFL2		
A0A0G2K5T4		
A6JHL5		
A6ICF5		
A0A8I5ZT35		
D3ZUT0		
A0A8I5ZWH4		
A0A8I5ZJD2		
A6I359		
A0A8I5ZRX6		
A6HHA2		
A6ICH4		
A0A0G2K1U4		
A0A0G2K744		
A0A8I6AFI9		
A0A8I6A4D5		
A0A0G2JZT1		
A0A0G2JT65		
A0A0G2KA68		
F1LXN4		
A6I579		
A0A0G2JVZ7		
A0A0G2K0J0		
D5MTH0		
A0A8I6GL06		
A0A8I6A9F6		
A6IY89		
A0A8I5ZZN6		
A0A0G2JWP2		
A0A8I5Y6D9		
A0A0G2K4G0		
A0A8I6AMR5		
A0A8I5ZNP4		
A6I3X8		
A0A0G2K4V5		
F1M9Q3		
A0A8I5ZRB3		
A0A8I5ZQ25		
A6I1B7		
E9PSN4		
A0A8I6A4U2		

Biology Open • Supplementary information

A0A0G2K240		
A6KTE6		
Q499U7		
A0A0G2JZI9		
A0A0G2JZJ3		
A6K479		
A0A8I6A139		
D3ZPX4		
A6J9C1		
P25304		
P15390		
A6J639		
A0A8I6AKB3		
D4A518		
P23739		
D3ZWP6		
A0A096MJY0		
A0A8I5ZS58		
A0A8I5ZRT6		
A0A0G2K2T4		
A0A8I5ZW24		
A0A0G2K836		
A0A0G2JUN6		
A0A8I6A4J8		
D3ZES7		
A0A8I6A5J5		
D4A7F0		
A0A0G2JVM2		
A0A8I5ZXU5		
F1LWJ1		
D3Z981		
A0A8L2Q1J0		
A0A8I6GK80		
A0A8I5ZK89		
A0A8I6ACF8		
Q9JLH5		
A0A8I5ZNY8		
A0A8I6ABT2		
A0A0G2K089		
A0A8I5ZM19		
A0A8I5ZTM5		
A0A8I6A0T7		
A0A8I6A7A1		
A0A8I6AA98		
A0A8I6A2F7		
A0A8I5ZMH8		

Biology Open • Supplementary information

A6KG07		
A0A8I6AGG2		
A0A8I5ZUC3		
A0A8I5ZKA2		
A6KLR4		
A6IIJ5		
F1LTE0		
A0A0G2K0F5		
F1LNB9		
A6N7T8		
B6RK61		
A0A0G2K511		
A6HVW1		
A0A0U1RRZ5		
A0A8I6B1B9		
A6KFZ7		
A6IH94		
A0A0G2K1V4		
G3V8B0		
A0A0G2K484		
D3ZIT7		
A6I3U3		
Q5TKR9		
A6K938		
P02563		
A0A8I5ZNN9		
A0A8I6AFM0		
A0A8I6A687		
A6JAN1		
P12847		
A0A8I6GJQ5		
D3ZD79		
A0A0G2K237		
A0A8I6AL19		
A0A0G2K7F0		
O88420		
A0A8I6GJS5		
D4ABD7		
O08562		
Q62812		
A0A8I6GIU0		
A0A8I5ZNR8		
A6N7T7		
A0A0G2K867		
A0A0G2K207		
A0A8I5ZTX2		

Biology Open • Supplementary information

P04774		
Q9JLT0		
A6J281		
F1LNF0		
A0A096MJA7		
Q6TXG6		
A0A8I5ZN78		
A0A0G2K1L0		
A0A8L2UMC0		
A6HVF8		
A0A8I5YCF2		
A0A8I6A0R2		
A0A8I5Y064		
A6IRJ2		
A6KEX2		
A0A0G2JW88		
D3ZYN7		
D4AB99		
A0A8I6GLP8		
A0A8I5ZNR1		
A0A0G2K9H4		
A0A8I5ZM58		
A0A8I5Y7C8		
A0A8I6A6U4		
A6JFK0		
A0A140TAJ5		
A0A0G2JV34		
F1LSM8		
A0A8I6GIY0		
A0A0G2K481		
D3ZXQ5		
A0A0G2JV16		
A6J3T0		
A0A8I6A006		
A0A8I6AWF1		
A0A8I5ZMK5		
A0A8I5YBG1		
A0A8I5ZKQ3		
A6KN33		
A0A0G2K3A7		
A0A0G2K175		
A6IIN6		
A0A8I6GKZ6		
A0PJ39		
D3ZRB5		
A0A8I6GLS2		

A0A8I6AJE2		
Q9Z0Y8		
A0A140UHX6		
A6JKW5		
A6I6C4		
A0A096MKF8		
A0A8I6ATU8		
A6JQM3		
E9PT50		
A0A8I6GHM0		
A0A8I6A538		
A0A8I6GDM7		
A0A8I6AVE6		
A6HPH8		
A6IRA3		
A0A8I5ZLL1		
A0A8I5ZM44		
A0A0G2K8H2		
F1MA36		

Biology Open • Supplementary information

Table S2. LC-MS parameters for EPF

LC Method Conditions

Buffer A	0.1% Formic Acid in MS Grade water
Buffer B	0.1% Formic Acid in ACN
Run Time	60 mins
Flow Rate	0.30 mL/min
Injection Volume	8.0 µL
Column	Acquity UPLC BEH Peptide C18, 150 X 2.1, 1.7µ, 300A°

Time	% A	% B
Initial	98 .0	2
5	98	2
47	50	50
52	0	100
56	0	100
56.1	98	2
60	98	2

MS Method Conditions

Ionization Mode	Positive
Scan Range	120-2500 m/z
Capillary Voltage	3 kV

Source Temperature	100ºC
Desolvation Gas Flow	900 L/Hr
Desolvation Temperature	300ºC
Cone Voltage	25V
Reference lock Mass Compound	Leucine Enkephalin

Analysis Parameters:

Software	PLGS (Protein Lynx Global Server 3.0.2)
Database	*Rattus norvegicus*

Workflow parameters:

Peptide tolerance	30ppm
Fragment Tolerance	100ppm
Min Fragment Ion Matches per protein	2
Min peptide Ion matches per protein	5
Fixed Modifier Reagents	Carbamidomethyl C
Variable Modifier Reagent	Oxidation
False Discovery rate	30
